# Case Report: Dyke-Davidoff-Masson syndrome resulting from a rare combination of hypoplastic left posterior cerebral artery and ipsilateral fetal-type posterior communicating artery

**DOI:** 10.3389/fnhum.2025.1629156

**Published:** 2025-09-09

**Authors:** He Huang, Chunyu Wang, Huijuan Hua, Yingju Zhang, Bo Zhao, Dongjun Wan

**Affiliations:** ^1^Department of Neurology, The 940th Hospital of Joint Logistics Support Force of the Chinese People’s Liberation Army, Lanzhou, China; ^2^Department of Endocrinology, The 940th Hospital of Joint Logistics Support Force of the Chinese People’s Liberation Army, Lanzhou, China

**Keywords:** Dyke-Davidoff-Masson syndrome, cerebral hemiatrophy, cerebrovascular disease, vascular malformation, cognitive impairment, epilepsy

## Abstract

**Introduction:**

Dyke-Davidoff-Masson syndrome (DDMS) is a rare neurological disorder characterized by unilateral hemiparesis, facial asymmetry, severe epilepsy, and intellectual disability. While congenital DDMS is predominantly attributed to anterior circulation anomalies [e.g., internal carotid artery (ICA) or middle cerebral artery (MCA) hypoplasia], posterior circulation involvement remains unreported. Here, we present the first documented case of congenital DDMS resulting from a rare combination of hypoplastic left posterior cerebral artery (PCA) and ipsilateral fetal-type posterior communicating artery (FTP).

**Case presentation:**

A 19-year-old male exhibited atypical DDMS manifestations: absence seizures, preserved motor function, and occipitotemporal cognitive deficits (MoCA: 20/30). Neuroimaging revealed classic DDMS features. Angiography confirmed left PCA hypoplasia with FTP persistence, while CT perfusion demonstrated chronic left PCA hypoperfusion. Lamotrigine (100 mg/day) and regular cognition rehabilitative training resulted in good symptom control.

**Conclusion:**

This case identifies PCA hypoplasia with FTP as a novel DDMS etiology, challenging the MCA/ICA-centric paradigm. The “posterior phenotype” (absence seizures, preserved motor function, occipitotemporal cognitive deficits) expands DDMS heterogeneity. Multimodal imaging (angiography/perfusion) is diagnostic gold-standard, while personalized therapy optimizes outcomes.

## Introduction

Dyke-Davidoff-Mason syndrome (DDMS) is a rare neurological disorder characterized predominantly by unilateral hemiparesis, facial asymmetry, severe epilepsy, and intellectual disability (Alam et al., 2018). Its typical neuroimaging features include atrophy of the unilateral hemisphere, enlargement of the ipsilateral lateral ventricle, and thickening of the ipsilateral cranial vault (Rondao et al., 2023; Zamora and Kontzialis, 2015). Etiologically, DDMS can be classified into congenital and acquired subtypes (Aguiar et al., 1998; Rondao et al., 2023). Previous studies have demonstrated that vascular anomalies leading to decreased cerebral blood flow supply are the main causes of congenital DDMS (Ayas et al., 2017; Gökçe et al., 2017; Rondao et al., 2023). However, to our best known, available congenital DDMS cases associated with definitive vascular anomalies occurring in anterior cerebral circulation [e.g., internal carotid artery (ICA) or middle cerebral artery (MCA)], whereas no case reports have been reported for unilateral posterior cerebral artery (PCA) (Afifi, 1987; Aggarwal et al., 2017; AlHatmi et al., 2023; Bagazgoitia et al., 2010; Bekci et al., 2016; Gökçe et al., 2017; Gul et al., 2024; Liao et al., 2018; Pinto et al., 2013; Ruggieri et al., 2012; Ruggieri et al., 2016; Sarikaya and Sarikaya, 2007; Sener and Jinkins, 1992; Stred et al., 1986; TEAL et al., 1973; Ünal et al., 2004; Yadav et al., 2009) (Supplementary Table 1).

Here, we present the first documented case of congenital DDMS in a man resulting from a rare combination of hypoplastic left PCA and ipsilateral fetal-type posterior communicating artery (FTP), with atypical manifestations (absence seizures, preserved motor function, occipitotemporal cognitive deficits). This finding provides novel evidence that contributes to the expansion of the spectrum of vascular etiology and the heterogeneity of clinical manifestations in DDMS.

## Case presentation

A 19-year-old, right-handed male was admitted to our department due to the occurrence of recurrent absence seizures over a period of 6 months. Initially, he had episodes of absence seizures that attacked two to three times per day and lasted for approximately 1 min. Gradually, his seizures became more severe, with approximately 6–7 attacks per day, each lasting 2–3 min. He was delivered via an uneventful caesarean section at a local hospital after a gestation of 39 weeks. There were no reported complications during pregnancy or the perinatal period, and no accidents have been noted in his medical history. The family history is unremarkable. Notably, his mother revealed that he displayed weaker learning abilities compared to peers starting at the age of 6. The notable co-occurrence of both learning disabilities and recurrent seizure in our patient prompted medical evaluation.

**Figure 1:**
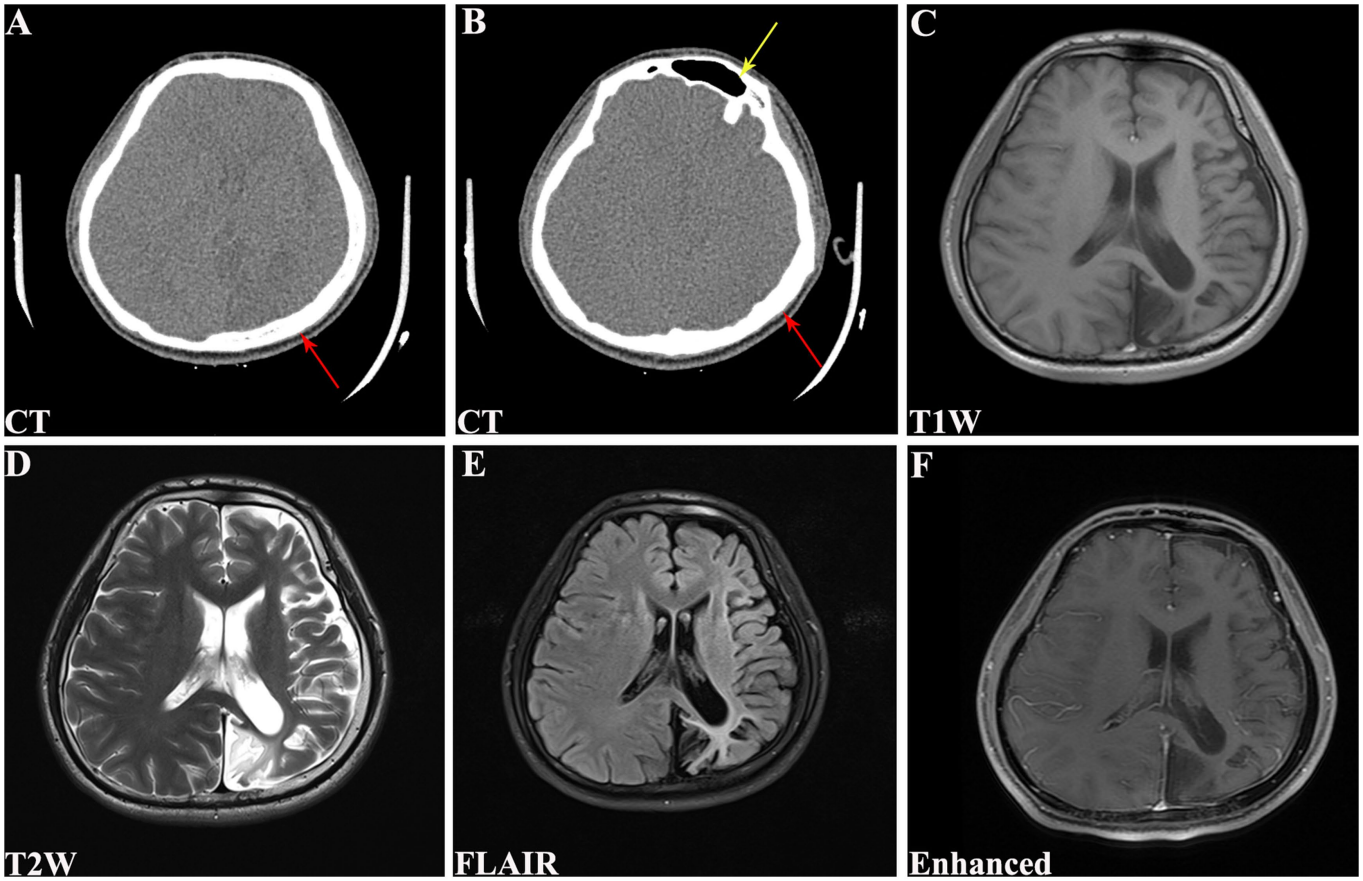
Cranial CT and MRI scans of the patient. **(A)** CT revealing left hemisphere atrophy with Compensated cranial thickening (red arrow). **(B)** CT demonstrating hyperpneumatization of the left frontal sinus (yellow arrow) with compensated cranial thickening (red arrow). **(C)** T1W, **(D)** T2W, **(E)** FLAIR, and **(F)** enhanced MRI showing left cerebral hemiatrophy along with enlargement of ipsilateral lateral ventricle. CT, computed tomography; MRI, magnetic resonance imaging; T1W, T1-weighted; T2W, T2-weighted; FLAIR, fluid attenuated inversion recovery.

**Figure 2:**
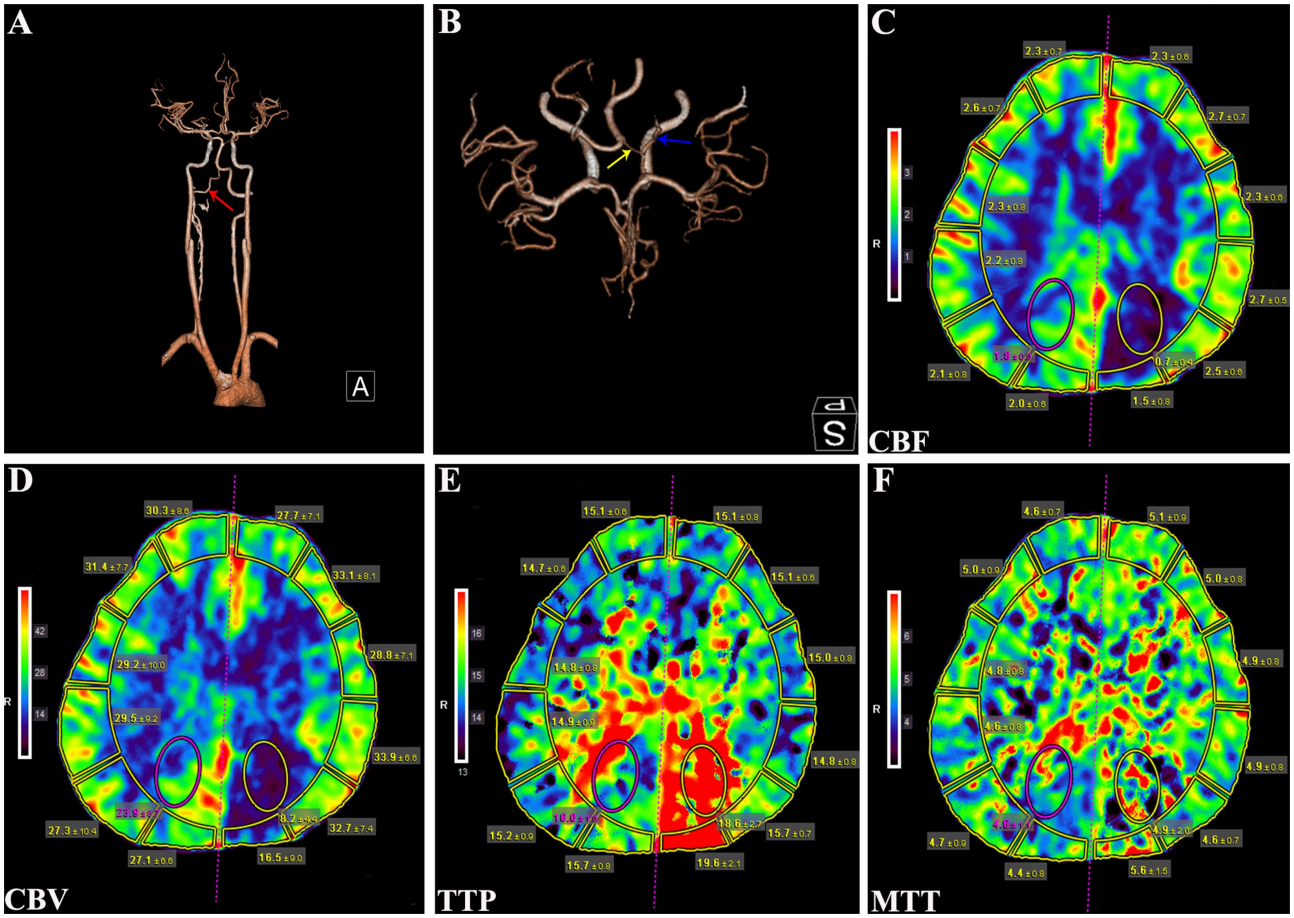
CTA and CTP analysis of the patient. **(A)** CTA showing a hypoplastic right VA (red arrow). **(B)** CTA revealing the left PCA originating from ipsilateral ICA with a hypoplastic P1 segment (yellow arrow) and a slenderly subsequent artery stem (blue arrow). **(C)** CBF, **(D)** CBV, **(E)** TTP, and **(F)** MTT parameters of CTP analysis demonstrating hypoperfusion in the left PCA supplying regions. CTA, computed tomography angiography; VA, vertebral artery; PCA, posterior cerebral artery; ICA, internal carotid artery; CBF, cerebral blood flow; CBV, cerebral blood volume; TTP, time to peak; MTT, mean transit time.

Physical examination revealed no remarkable abnormalities in our patient. The Montreal Cognitive Assessment (MoCA) yielded a score of 20 in our patient, suggesting mild cognitive impairment with predominant deficits in the language and delayed recall domains. The electroencephalogram showed interhemispheric asymmetry, increased activity of sharp slow complex waves in the left occipital and left middle-posterior temporal regions. Cranial computed tomography (CT) and magnetic resonance imaging (MRI) revealed left hemisphere atrophy (Figures 1A–F), thickening of the ipsilateral cranial vault (Figures 1A,B), dilatation of the ipsilateral frontal sinus (Figure 1B), and enlargement of the left ipsilateral ventricle (Figures 1C–F). Furthermore, CT angiography (CTA) showed hypoplasia of the right vertebral artery (VA), and revealed that the left PCA originated from the ipsilateral ICA with a hypoplastic P1 segment, a slender subsequent artery stem, and sparse branches (Figures 2A,B). Further CT perfusion imaging (CTP) analysis confirmed the areas supplied by the left PCA exhibited hypoperfusion (Figures 2C–F). Other items consisting of psychological assessment, magnetic resonance venography, ultrasonography of the heart and other internal organs, chest CT scan, blood routine test, blood biochemistry index, blood coagulation analysis, urinalysis, and autoimmune disease parameters, were within normal limits.

Based on the classical neuroimaging features, our patient was diagnosed with DDMS and received treatment with oral lamotrigine 50 mg twice daily, as well as regular cognition rehabilitative training. Since then, he has been regularly followed up by our department. During the 12-month follow-up period, the patient maintained complete seizure freedom (0 events/month) on lamotrigine monotherapy at a maintenance dose of 100 mg/day. Cognitive function demonstrated mild improvement (Supplementary Table 2).

## Discussion

This case is the first report of congenital DDMS caused by hypoplasia of left PCA combined with ipsilateral FTP, offering novel insights into the etiology and clinical phenotypes of this rare disorder. Traditional perspectives posit that congenital DDMS is mainly associated with vascular anomalies in anterior circulation anomalies (e.g., ICA or MCA), leading to unilateral cerebral hypoperfusion and secondary cerebral atrophy (Atalar et al., 2007; Rondao et al., 2023). Notably, isolated PCA anomalies have not been previously documented in this context. In the present case, neuroimaging revealed an anomalous left PCA origin from the ipsilateral ICA with P1 segment hypoplasia, resulting in territorial hypoperfusion. This vascular configuration suggests that chronic hemodynamic impairment during critical neurodevelopmental stages precipitated prolonged hemispheric hypoperfusion, ultimately inducing characteristic anatomical features of congenital DDMS—including ipsilateral cerebral atrophy, progressive ventriculomegaly, and compensatory calvaria thickening (Bhol et al., 2021; Zilkha, 1980).

The coexistence of FTP further underscores the role of Will’s circle variations in the pathogenesis of congenital DDMS. FTP, a common anatomical variant that occurs in 20–30% of the general population, has historically been considered benign (Davidoiu et al., 2023). However, recent studies indicate that FTP is strongly associated with increased risk of cerebrovascular injury, such as white matter hyperintensities, lacunar infarcts and posterior circulation infarctions (Feng et al., 2023; Hsu et al., 2021; Mann et al., 2021). In this case, FTP likely aggravated hypoperfusion in the left PCA-supplied area by impairing the posterior–anterior collateral circulation (Srichawla and Garcia-Dominguez, 2024). This finding not only expands the spectrum of vascular etiologies in congenital DDMS, but also proposes a unique pathogenic pattern combining PCA hypoplasia with ipsilateral FTP.

Notably, this case demonstrates a distinct cognitive profile: whereas profound intellectual disability typically manifests in childhood among classic DDMS patients (Ayas et al., 2017; Rondao et al., 2023), our patient exhibited relatively preserved overall cognition, with deficits selectively localized to language and delayed recall domains. This distinct cognitive phenotype may be attributed to the following pathophysiological mechanisms: (I) vascular sparing of anterior circulation networks preserved prefrontal-mediated executive functions (working memory/attention), contrasting with impaired posterior vascular territories (Park et al., 2011; Popplau and Hanganu-Opatz, 2024); (II) left PCA territory ischemia directly disrupting occipito-temporal hubs critical for language processing (lingual/fusiform gyri) (Cook et al., 2014; Papagno et al., 2023), consistent with verbal fluency deficits observed here and in left PCA infarct patients (90.1% verbal memory impairment in left PCA vs. 71% in right PCA) (Benke et al., 2022); (III) chronic hippocampal hypoperfusion via compromised PCA-temporal branches, inducing subclinical neuronal loss and selective recall deficits without global amnesia—mirroring extrahippocampal lesion effects in PCA stroke (Benke et al., 2022; Watanabe et al., 2019); (IV) neuroimaging did not reveal any cerebral penetrating malformations, suggesting that the hypoperfusion event may have occurred in the late gestational or perinatal period, when neuroplasticity allowed synaptic reorganization and network remodeling to partially compensate for the ischemic damage (Rondao et al., 2023). Collectively, this case delineates a vascular-territory-specific cognitive phenotype in congenital DDMS, where left PCA hypoperfusion targets: (a) language networks, (b) hippocampal-thalamic pathways, while sparing (c) frontal-executive domains.

Moreover, a recent systematic review consisting 188 DDMS cases revealed that motor deficits (hemiparesis in 97.6% and facial asymmetry in 100% of congenital DDMS patients) and generalized tonic–clonic seizures represent hallmark clinical features (Rondao et al., 2023). However, this case demonstrated marked phenotypic divergence, exhibiting neither hemiparesis nor facial asymmetry. The predominant epileptic manifestation was characterized by atypical absence seizures. This discrepancy can be attributed to: (I) the ischemic damage was strictly limited to the areas supplied by the PCA (occipital and temporal lobes), without involving the motor system (Xalxo et al., 2025); and (II) the focal injury to the left temporal-occipital lobe may have triggered absence seizures through abnormal synchronized discharges of the temporal-occipital and thalamocortical networks (Danielson et al., 2011; Groulx-Boivin et al., 2024; Kumar et al., 2023). Interestingly, according to the literature search, only one previous case of DDMS was reported to have absence seizures (Parker et al., 1972). The present case represents a notable exception, as it is the second documented instance of DDMS associated with such an atypical epilepsy phenotype.

This case also highlights three important implications for clinical practice: first, attention should be paid to atypical DDMS manifestations even in the absence of hemiparesis and facial asymmetry, and the possibility of DDMS should be considered when the patient presents with mild cognitive impairment and atypical epilepsy (e.g., absence seizures) to avoid underdiagnosis. Second, characteristic neuroimaging findings remain the gold-standard criteria for DDMS (Durcan et al., 2018; Zamora and Kontzialis, 2015). High-resolution MRA or CTA, combined with perfusion studies, is essential to delineate cerebrovascular abnormalities and guide prognosis. In addition, combining multidisciplinary disciplines such as neuroimaging, epilepsy specialties, and neurorehabilitation to develop personalized therapeutic regimens may improve symptom control and functional outcomes.

This report is limited by the absence of definitive prenatal imaging to confirm the congenital etiology of DDMS, which introduces diagnostic uncertainty. While the developmental trajectory and radiological characteristics strongly support a congenital origin, future studies documenting early-stage imaging would be required to establish causal relationships. Consequently, this case represents presumed congenital DDMS rather than a definitively proven congenital anomaly.

In conclusion, this report identifies for the first time that unilateral PCA hypoplasia with ipsilateral FTP as a novel etiology of congenital DDMS, challenging the traditional cognitive framework that considers MCA/ICA developmental abnormalities as the core etiology of congenital DDMS. The unique clinical triad (absence seizures, the absence of motor deficits, and mild cognitive impairment) expands the phenotypic spectrum of DDMS. Neuroimaging in combination with angiography is key to a definitive diagnosis, while personalized antiepileptic therapy and cognitive rehabilitation can effectively improve patients’ quality of life.

## Data Availability

The original contributions presented in the study are included in the article/Supplementary material, further inquiries can be directed to the corresponding authors.
