# Supplementary material for: Case Report: Dyke-Davidoff-Masson syndrome resulting from a rare combination of hypoplastic left posterior cerebral artery and ipsilateral fetal-type posterior communicating artery
Source: Front Hum Neurosci. 2025 Sep 9;19:1629156. doi: 10.3389/fnhum.2025.1629156 (PMC12455352; doi:10.3389/fnhum.2025.1629156)
Supplement: Supplementary file 1 [file Table_1.DOCX]

***Supplementary Material***

**Supplementary table 1** Demographic and clinicoradiological findings of cases with congenital Dyke–Davidof–Masson syndrome

| Case | Age | Gender | HA | Major clinical findings | Definite vascular etiologies^*^ | Ref |
| --- | --- | --- | --- | --- | --- | --- |
| 1 | 41 years | M | L | Seizure, intellectual disability, hemiparesis (R), facial asymmetry (L) | Congenital absence of left ICA | (TEAL et al., 1973) |
| 2 | 66 years | F | L | Seizure, facial asymmetry (L) | Congenital absence of left ICA | (TEAL et al., 1973) |
| 3 | 6.5 months | M | L | Motor disability, arm monoparesis (R), upper extremity hypertension (R), facial asymmetry (L) | Congenital coarctation of the mid-aortic arch | (Stred et al., 1986) |
| 4 | 4 months | F | L | Seizure, hemiparesis (R), facial asymmetry (L) | Congenital hypoplasia of left ICA | (Afifi, 1987) |
| 5 | 14 years | M | R | Seizure, hemiparesis (L), facial asymmetry (R) | Congenital hypoplasia of right MCA | (Sener and Jinkins, 1992) |
| 6 | 2 years | M | L | Seizure, hemiparesis (R), facial asymmetry (L) | Congenital hypoplasia of left MCA | (Sener and Jinkins, 1992) |
| 7 | - | - | L | - | Congenital occlusion of left MCA | (Ünal et al., 2004) |
| 8 | 36 years | F | L | Intellectual disability, facial asymmetry (L), hemiparesis (R) | Congenital occlusion of left ICA | (Sarikaya and Sarikaya, 2007) |
| 9 | 14 hours | M | L | Seizure | Congenital hypoplasia of left ICA | (Yadav et al., 2009) |
| 10 | 2 years | F | R | Seizure, intellectual disability, facial asymmetry (R), hemiparesis (L), skin vascular malformation on face (R) | Congenital hypoplasia of right WC | (Bagazgoitia et al., 2010) |
| 11 | 2.5 years | M | R | Seizure, intellectual disability, facial asymmetry (R), hemiparesis (L), skin vascular malformation on face and over entire body (B) | Congenital hypoplasia of right WC | (Ruggieri et al., 2012) |
| 12 | 12 years | F | L | Headache, tinnitus, hemiparesis (L), facial asymmetry (L), hearing loss (R), skin vascular malformation on face (L) | Congenital hypoplasia of left WC | (Ruggieri et al., 2012) |
| 13 | 2 months | F | L | Intellectual disability, facial asymmetry (L), hemiparesis (R) | Congenital hypoplasia of left MCA | (Piro et al., 2013) |
| 14 | 7 years | M | R | Seizure, intellectual disability, facial asymmetry (R), hemiparesis (L), skin vascular malformation on face (L) and body (L) | Congenital hypoplasia of right WC | (Ruggieri et al., 2016) |
| 15 | 12 years | M | R | Seizure, intellectual disability, facial asymmetry (R), skin vascular malformation on face (B) | Congenital hypoplasia of right WC | (Ruggieri et al., 2016) |
| 16 | 34 years | M | R | Seizure, facial asymmetry (R), hemiparesis (L), skin vascular malformation on face (B) | Congenital hypoplasia of right WC | (Ruggieri et al., 2016) |
| 17 | 8 years | M | L | Seizure, blindness (L), hemiparesis (R), facial asymmetry (L), skin vascular malformation on face (L) | Congenital hypoplasia of left MCA | (Bekci et al., 2016) |
| 18 | 15 years | F | L | Seizure, intellectual disability, hemiparesis (R), facial asymmetry (L) | Congenital hypoplasia of left MCA | (Gökçe et al., 2017) |
| 19 | 29 weeks’ gestational age | M | L | - | Congenital absence of left ACA and MCA | (Aggarwal et al., 2017) |
| 20 | 26 years | M | L | Seizure, intellectual disability, hemiparesis (R), facial asymmetry (L) | Congenital absence of left MCA | (Liao et al., 2018) |
| 21 | 42 years | M | L | Seizure, intellectual disability, facial asymmetry (R), hemiparesis (R), circumduction gait | Congenital hypoplasia of left MCA | (AlHatmi et al., 2023) |
| 22 | 13 months | F | L | Hemiplegia (R) | Congenital hypoplasia of left MCA | (Gul et al., 2024) |

M: male; F: female; R: right; L: left; B: both sides; HA: hemisphere atrophy; ICA: internal carotid artery; MCA: middle cerebral artery; ACA: anterior cerebral artery; WC: Will’s circle; Ref: references; “-”: not mentioned in the case; “*”: vascular abnormalities were confirmed by magnetic resonance angiography (MRA), computerized tomography angiography (CTA), or digital subtracted angiography (DSA).

**References**

Afifi, A.K. (1987). Cerebral hemiatrophy, hypoplasia of internal carotid artery, and intracranial aneurysm. Archives of Neurology 44, 232. doi: 10.1001/archneur.1987.00520140090024.

Aggarwal, A., Aggarwal, A.K., Kapoor, A., Kapoor, R., and Bansal, A. (2017). Hemiatrophy of brain: antenatal ultrasonography and mri/postnatal mri diagnosis with the introduction of “shifted falx sign”. J Med Ultrason (2001) 44, 147-151. doi: 10.1007/s10396-016-0744-7.

AlHatmi, A., Almashaikhi, T., and Ajmi, E.A. (2023). Imaging features of dyke-davidoff-masson syndrome. Sultan Qaboos University Medical Journal 23, 122-124. doi: 10.18295/squmj.9.2022.055.

Bagazgoitia, L., García-Peñas, J.J., Duat-Rodríguez, A., Hernández-Martín, Á., and Torrelo, A. (2010). Facial capillary malformation and dyke-davidoff-masson syndrome. Pediatr Neurol 43, 202-204. doi: 10.1016/j.pediatrneurol.2010.04.011.

Bekci, T., Bilgici, M.C., Turgut, E., and Aslan, K. (2016). A rare combination: sturge–weber syndrome and accompanying dyke–davidoff–masson syndrome. Acta Neurol Belg 116, 79-81. doi: 10.1007/s13760-015-0511-3.

Gökçe, E., Beyhan, M., and Sade, R. (2017). Radiological imaging findings of dyke–davidoff–masson syndrome. Acta Neurol Belg 117, 885-893. doi: 10.1007/s13760-017-0778-7.

Gul, E., Atalar, M.H., and Atik, I. (2024). Evaluation of the contralateral hemisphere with dwi in pediatric patients with dyke–davidoff–masson syndrome. Acta Neurol Belg 124, 911-918. doi: 10.1007/s13760-024-02473-5.

Liao, Y., Wu, C., and Wang, J. (2018). A case report of dyke-davidoff-masson syndrome. Chin J Neurol 51, 60-61. doi: 10.3760/cma.j.issn.1006-7876.2018.01.013.

Piro, E., Piccione, M., Marrone, G., Giuffrè, M., and Corsello, G. (2013). Dyke-davidoff-masson syndrome: case report of fetal unilateral ventriculomegaly and hypoplastic left middle cerebral artery. Ital J Pediatr 39, 32. doi: 10.1186/1824-7288-39-32.

Ruggieri, M., Milone, P., Pavone, P., Falsaperla, R., Polizzi, A., Caltabiano, R., Fichera, M., Gabriele, A.L., Distefano, A., De Pasquale, R., Salpietro, V., Micali, G., and Pavone, L. (2012). Nevus vascularis mixtus (cutaneous vascular twin nevi) associated with intracranial vascular malformation of the dyke–davidoff–masson type in two patients. Am J Med Genet A 158A, 2870-2880. doi: 10.1002/ajmg.a.35221.

Ruggieri, M., Polizzi, A., Strano, S., Schepis, C., Morano, M., Belfiore, G., Palmucci, S., Foti, P.V., Pirrone, C., Sofia, V., David, E., Salpietro, V., Mankad, K., and Milone, P. (2016). Mixed vascular nevus syndrome: a report of four new cases and a literature review. Quant Imaging Med Surg 6, 515-524. doi: 10.21037/qims.2016.10.09.

Sarikaya, B., and Sarikaya, S. (2007). Dyke–davidoff–masson syndrome revisited: a didactic case with interesting imaging findings. Australasian Radiology 51, B10-B13. doi: 10.1111/j.1440-1673.2007.01834.x.

Sener, R.N., and Jinkins, J.R. (1992). Mr of craniocerebral hemiatrophy. Clin Imaging 16, 93-97. doi: 10.1016/0899-7071(92)90119-T.

Stred, S.E., Byrum, C.J., Bove, E.L., and Oliphant, M. (1986). Coarctation of the midaortic arch presenting with monoparesis. The Annals of Thoracic Surgery 42, 210-212. doi: 10.1016/S0003-4975(10)60522-X.

TEAL, J.S., RUMBAUGH, C.L., BERGERON, R.T., and SEGALI, H.D. (1973). Congenital absence of the internal carotid artery associated with cerebral hemiatrophy, absence of the external carotid artery, and persistence of the stapedial artery. Ajr Am J Roentgenol 118, 534-545. doi: 10.2214/ajr.118.3.534.

Ünal, Ö., Tombul, T., Çırak, B., Anlar, Ö., İncesu, L., and Kayan, M. (2004). Left hemisphere and male sex dominance of cerebral hemiatrophy (dyke–davidoff–masson syndrome). Clin Imaging 28, 163-165. doi: 10.1016/S0899-7071(03)00158-X.

Yadav, P., Kumar, A., Saili, A., and Datta, V. (2009). Congenital hypoplasia of the internal carotid artery. The Indian Journal of Pediatrics 76, 1061-1062. doi: 10.1007/s12098-009-0203-1.
