# Supplementary material for: Case Report: Dyke-Davidoff-Masson syndrome resulting from a rare combination of hypoplastic left posterior cerebral artery and ipsilateral fetal-type posterior communicating artery
Source: Front Hum Neurosci. 2025 Sep 9;19:1629156. doi: 10.3389/fnhum.2025.1629156 (PMC12455352; doi:10.3389/fnhum.2025.1629156)
Supplement: Supplementary file 2 [file Table_2.DOCX]

***Supplementary Material***

**Supplementary table 2** Longitudinal evolution of seizure frequency and cognitive function in the patient

| Timepoint | Seizure Frequency  (events/month) | MoCA Score (/30) | Intervention Phase |
| --- | --- | --- | --- |
| Baseline | 195 | 20 | Pre-treatment |
| 3-month follow-up | 30 | 21 | Lamotrigine + Rehabilitation |
| 6-month follow-up | 3 | 22 | Maintenance therapy |
| 9-month follow-up | 0 | 22 | Maintenance therapy |
| 12-month follow-up | 0 | 22 | Maintenance therapy |
